# Ethnic Disparity in the Incidence of Scoliosis Among Adolescents in Tianzhu Tibetan Autonomous County, China

**DOI:** 10.3389/fpubh.2022.791550

**Published:** 2022-04-27

**Authors:** Haibin Guo, Nan Chen, Yuqi Yang, Xuan Zhou, Xin Li, Yan Jiang, Jiaoling Huang, Qing Du

**Affiliations:** ^1^Department of Rehabilitation Medicine, Xinhua Hospital Affiliated to Shanghai Jiao Tong University School of Medicine, Shanghai, China; ^2^College of Global Public Health, New York University, New York, NY, United States; ^3^Department of Rehabilitation Medicine, Third People's Hospital of Gansu Province, Lanzhou, China; ^4^School of Public Health, Shanghai Jiao Tong University School of Medicine, Shanghai, China

**Keywords:** scoliosis, ethnic disparity, socioeconomic status, risk factors, hukou

## Abstract

**Objectives:**

To determine the influence of ethnic disparities, socioeconomic status (SES) and hukou on the incidence of scoliosis.

**Methods:**

We enrolled 2,445 junior high school students (Han: 1,153; ethnic minorities: 1,292) aged 12–16 years from two schools in Tianzhu Tibetan Autonomous County, Gansu Province from November 2020 to February 2021. We measured the angle of trunk rotation (ATR) using a scoliometer. Two-factor analysis of variance was used to comparatively analyze differences in the ATR according to ethnicity, age, sex, body mass index (BMI), and SES between the groups. Associations between risk factors and scoliosis were estimated using odds ratios and 95% confidence intervals with an unconditional multivariate logistic regression model for the two groups.

**Results:**

Adolescents with Han ethnicity were more likely to have scoliosis than their ethnic minority counterparts (10.8% vs. 7.1%, *P* < 0.05). The ATR value in the Han group decreased with age whereas the minority group showed an upward trend (*P* < 0.05). The difference between ethnic groups was not significant, only at level 3. In particular, the ATR values among Han girls were significantly higher than those of ethnic minority girls (*P* < 0.05). Compared with Han adolescents, the BMI of ethnic minorities had a greater impact on the ATR. A statistically significant difference in SES was found between the two ethnic groups (*P* < 0.05). Hukou and parents' occupation had an important influence on the onset of scoliosis.

**Conclusions:**

Han adolescents had higher ATR values and were more likely to have scoliosis than ethnic minority adolescents in our study. Growth and development indicators (height and BMI) and differences in SES between the two groups played an important role.

## Introduction

Scoliosis is the most common pediatric musculoskeletal disorder, which causes three-dimensional spinal deformity accompanied by lateral curvature of the spine not less than 10° and vertebral rotation. Idiopathic scoliosis (IS) accounts for 80% of all cases of scoliosis and affects children of all ethnicities and levels of socioeconomic status (SES) (1). The prevalence of IS is 2–3% according to epidemiological studies (2, 3). Previous long-term studies of IS present a poor prognosis, perpetuating the common misconception that IS inevitably leads to disability from back pain and cardiopulmonary compromise if patients do not receive early treatment (4, 5). Scoliosis is common in adolescence, especially among adolescent girls. The American Academy of Orthopedic Surgeons recommends screening girls at age 11 years and again at age 13 years and screening boys once at 13–14 years of age. Most doctors are committed to early detection and recommend school scoliosis screening (SSS) (6). In recent years, increasingly more countries have begun to conduct SSS, such as Canada, the United States, Japan, and China (Hong Kong) (7). The American Academy of Pediatrics has recommended scoliosis screening using the Adam's forward bending test at routine health visits at ages 10, 12, 14, and 16 years (6).

According to existing reports, growth and development, living habits, SES, cultural differences, and even genes differ among different ethnic groups (8–13). Ethnic minorities often live in remote frontier areas and have a relatively small population size. In China, tremendous social transitions have enlarged the disparities between populations in the eastern and western parts of the country (14). It is unclear whether differences exist in the incidence of scoliosis between Han Chinese and ethnic minorities living in western China. Past research has mainly concentrated on eastern regions, such as Shanghai and Guangdong, where the prevalence of IS varies from 0.11% to 2.64% (15); however, few studies are available in western areas of China. At present, only one article in 2005 reported that the prevalence of scoliosis was 0.66% among 15,377 primary school students aged 6–13 years in Baiyin City, Gansu Province (16). Starting in 2019, the National Health Commission of China included abnormal spinal curvature as an early detection indicator in the monitoring of common diseases and health-influencing factors among students nationwide.

This study was conducted in Tianzhu Tibetan Autonomous County, a typical multiethnic settlement in Gansu Province, which is famous for animal husbandry. Ethnic minorities account for 37.1% of the total population, and Tibetans account for 97.14% of the ethnic minority population. It is located at the intersection of the Qinghai–Tibet Plateau, Loess Plateau, and Inner Mongolia Plateau, in an area known as the “Eye of Qinghai–Tibet.” Owing to the characteristics of local ethnic groups, its geography, and activities of animal husbandry, Tianzhu is a valuable area in epidemiological research.

In this study, we investigated adolescents from two representative ethnic groups in Tianzhu Tibetan Autonomous County to explore outcome disparities for scoliosis and to (1) present epidemiological findings according to the ethnicity of included adolescents; (2) assess differences in the magnitude of trunk asymmetry between adolescents with Han ethnicity and ethnic minority adolescents; and (3) observe risk factors of IS that should be considered in SSS.

## Materials and Methods

### Study Design

In this study, we included junior high school students as the study population. Prior to the start of screening, we calculated the sample size. According to N = Z^2^ × (P × (1–P))/E^2^; *N* = sample size, *Z* = 1.96 with 95% confidence interval, *P* = 4.3% probability (according to existing research in China (17)), and *E* = 1% margin of error. A sample of size of *N* = 1,580 was obtained. To prevent loss to follow up, we randomly selected two of 10 junior high school from which we enrolled a total of 2,510 students in this study (Han: 1,188 and ethnic minorities: 1,322; nearly 50% of junior high school students in Tianzhu). All participants signed an informed consent form. Consent from a parent or guardian was also required to participate in this study.

### Measurements

We designed a screening form that included basic information of participants, such as school, class, sex, age, height, and weight. We also included questions addressing SES such as parents' education level (classified into five groups: illiterate, primary school, middle school, senior high school, and university and above), parents' occupation (herder and non-herder), annual household income (classified into four groups: <30,000 RMB, 30,000–50,000 RMB, 50,000–80,000 RMB, and >80,000 RMB), hukou (urban residence and rural residence), and ethnicity (Han and ethnic minority), according to local multiethnic characteristics. We measured the angle of trunk rotation (ATR) using a scoliometer. All data were entered into EpiData on the same day. Quality control procedures were set up to ensure that the data entry was performed correctly. We found that 65 pieces of data had multiple or missing information for SES variables. Finally, 2,445 adolescents were included in this study.

### SSS and Criteria

Screening was carried out in accordance with the method described by Adamczewska et al. (18). Measurement was performed with participants in the Adam's forward bending test position at three levels: level 1, proximal thoracic (T1–T4); level 2, main thoracic (T5–T12); and level 3, lumbar (T12–L4) (18). In this test, direct visual observation is used to detect any abnormalities in the spinal curvature, such as a hump or asymmetry of the back. The Adam's forward bending test is easy to carry out, non-invasive, and safe. All screening examiners were uniformly trained before participating in SSS. In this study, ATR ≥ 5° was used as the criterion for a positive test result. According to guidelines of the International Scientific Society on Scoliosis Orthopaedic and Rehabilitation Treatment (SOSORT) (19), ATR <5° is considered normal and ATR ≥ 5° is considered positive for scoliosis. China has its own set of screening standards (GB/T 16133-−2014. Screening of spinal curvature abnormality of children and adolescents) (20): ATR <5° is considered normal, and 5° ≤ ATR <7°, 7° ≤ ATR <10°, and ATR ≥ 10° are considered degrees I, II, and III, respectively. Therefore, students with ATR ≥ 5° were recommended for whole spine erect anteroposterior projection X-ray at standing position in follow up.

### Statistical Analysis

All statistical analysis was performed using IBM SPSS 25.0 (IBM Corp., Armonk, NY, USA) and GraphPad Prism 8.0 (GraphPad Software, San Diego, CA, USA). Descriptive analyses were conducted to capture the sample characteristics, including frequency, percentage, mean, and standard deviation. We used the Mann–Whitney U test to analyze the average values of measurement items between groups. According to different screening standards, the chi-square test was used to analyze the differences in SSS results between the two groups. Two-factor analysis of variance was used to comparatively analyze differences in ATR according to age, sex, BMI, and SES between the groups. We also used an unconditional multivariate logistic regression model to analyze the risk factors of scoliosis, with odds ratios (ORs) and 95% confidence intervals (CIs). All *P*-values were two-tailed and the significance level was 0.05.

## Results

### Demographics

A total of 2,445 adolescents were enrolled in this study, of which 1,153 were Han (47.2%) and 1,292 (52.8%) were ethnic minorities; Zang accounted for nearly all minority adolescents (Zang, 91.9%; other, 8.1%). The average age and sex ratios were similar between the two groups. The parents of adolescents in both groups mainly had a primary school and junior high school education level, with more Han parents having a junior high school education and fewer having a university degree or above than the parents of minority adolescents. More parents of ethnic minority students worked as herders than those of Han students (see Table 1).

**Table 1:** Demographics and SES of families included in the study.

	**Han (*N* = 1,153) Mean ± SD/N (%)**	**Minority** **(*N* = 1,292)** **Mean ± SD/N (%)**	***p*-value**
Age (years)	14.08 ± 0.94	14.16 ± 0.96	0.058
Sex, Male (%)	580 (50.3)	675 (52.2)	0.338
Mass (kg)	50.56 ± 9.55	51.59 ± 9.58	0.004^**^
Height (cm)	162.42 ± 8.25	163.19 ± 8.61	0.016^*^
BMI (kg/cm^2^)	19.13 ± 3.16	19.33 ± 3.09	0.063
Hukou			0.379
Rural residents	990 (85.9)	1,093 (84.6)	
Urban residents	163 (14.1)	199 (15.4)	
Father's Education			0.017^*^
Illiterate	13 (1.1)	26 (2.0)	
Primary school	359 (31.1)	412 (31.9)	
Junior high school	517 (44.8)	532 (41.2)	
Senior high school	150 (13.0)	156 (12.1)	
University degree or above	104 (9.0)	161 (12.5)	
Missing	10 (0.9)	5 (0.4)	
Mother's Education			<0.0001^**^
Illiterate	11 (1.0)	27 (2.1)	
Primary school	358 (31.0)	438 (33.9)	
Junior high school	551 (47.8)	543 (42.0)	
Senior high school	126 (10.9)	120 (9.3)	
University degree or above	94 (8.2)	156 (12.1)	
Missing	13 (1.1)	8 (0.6)	
Father's Occupation (Herders)			0.001^**^
Yes	31 (2.7)	70 (5.4)	
No	1,113 (96.5)	1,218 (94.3)	
Missing	9 (0.8)	4 (0.3)	
Mother's Occupation (Herders)			0.015^*^
Yes	27 (2.3)	53 (4.1)	
No	1,121 (97.2)	1,234 (95.5)	
Missing	5 (0.4)	5 (0.4)	
Household income			0.589
<30,000 RMB	741 (64.3)	851 (65.9)	
30,000 to 50,000 RMB	311 (27.0)	338 (26.2)	
50,000 to 80,000 RMB	83 (7.2)	91 (7.0)	
>80,000 RMB	10 (0.9)	6 (0.5)	
Missing	8 (0.7)	6 (0.5)	
ATR (°)
Level 1	1.59 ± 1.07	1.54 ± 1.02	0.386
Level 2	2.06 ± 1.39	2.01 ± 1.37	0.405
Level 3	2.07 ± 1.49	1.95 ± 1.38	0.085
Max	2.73 ± 1.47	2.62 ± 1.42	0.095

*Data are n (%) or mean ± standard deviation (SD) unless otherwise specified. Level 1, proximal thoracic (T1–T4); level 2, main thoracic (T5–T12); level 3, lumbar (T12–L4)*.

* *p < 0.05*,

** *p < 0.01*.

*SES, socioeconomic status; BMI, body mass index; ATR, angle of trunk rotation*.

From Table 2, we can see that according to the two screening standards used in this study, there were significant differences between the groups. The positive rate of IS in the Han group was significantly higher than that in the minority group (maximum value among the three levels [max]: Han, 10.8%; minority, 7.1%). According to the Chinese standard, the proportion of Han adolescents with degree I (8.1%) was higher than that among minority adolescents (4.5%). The proportion of each degree at levels 2 and 3 also differed between the two groups.

**Table 2:** ATR values of the study population using two IS standards.

	**Region**	**Chinese standard** ^**a**^					**International standard** ^**b**^		
**ATR**		**(0,5)**	**(5,7)**	**(7,10)**	**10**~****	**Chi-square**	**(0,5)**	**5**~****	**Chi-square**
Level 1	Han	1,134 (98.4)	19 (1.6)	-	-	3.233	1,134 (98.4)	19 (1.6)	0.724
	Minority	1,276 (98.8)	14 (1.1)	2 (0.2)	-		1,276 (98.8)	16 (1.2)	
Level 2	Han	1,087 (94.3)	54 (4.7)	12 (1.0)	-	7.796^*^	1,087 (94.3)	66 (5.7)	2.482
	Minority	1,236 (95.7)	38 (2.9)	15 (1.2)	3 (0.2)		1,236 (95.7)	56 (4.3)	
Level 3	Han	1,076 (93.3)	54 (4.7)	23 (2.0)	-	10.333^*^	1,076 (93.3)	77 (6.7)	7.502^**^
	Minority	1,238 (95.8)	37 (2.9)	15 (1.2)	2 (0.2)		1,238 (95.8)	54 (4.2)	
Max	Han	1,029 (89.2)	93 (8.1)	31 (2.7)	-	17.401^**^	1,029 (89.2)	124 (10.8)	9.989^**^
	Minority	1,200 (92.2)	58 (4.5)	30 (2.3)	4 (0.3)		1,200 (92.9)	92 (7.1)	

*Data are n (%) or mean ± standard deviation unless otherwise specified*.

*Level 1, proximal thoracic (T1–T4); level 2, main thoracic (T5–T12); level 3, lumbar (T12–L4)*.

a *GB/T 16133-−2014. Screening of spinal curvature abnormality of children and adolescents*.

b *Guidelines of the International Scientific Society on Scoliosis Orthopaedic and Rehabilitation Treatment (SOSORT)*.

* *p < 0.05*,

** *p < 0.01*.

*IS, idiopathic scoliosis; ATR, angle of trunk rotation*.

**Figure 1:**
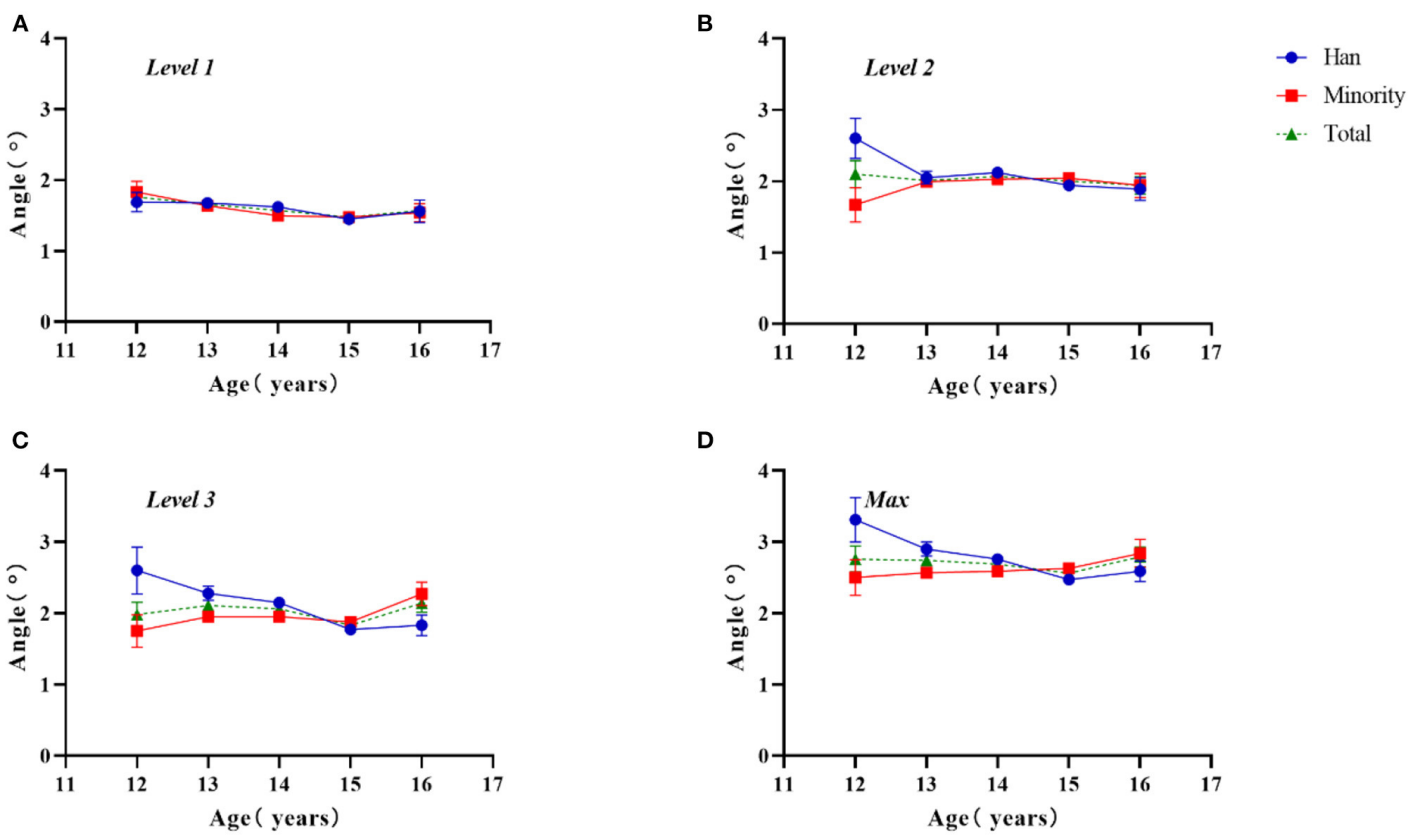
Two-factor analysis of variance to assess differences in ATR values by ethnicity and age. **(A)** Level 1, proximal thoracic (T1–T4); **(B)** level 2, main thoracic (T5–T12); **(C)** level 3, lumbar (T12–L4); **(D)** Max, maximum value among the three levels.

As shown in Figure 1, the ATR value at different levels varied with age between the two groups. At level 1, the two groups showed the same trend; both groups showed an increase in ATR with age, and the ATR value decreased (*F* = 3.528, *P* = 0.007). At level 3, the ATR value in Han adolescents decreased with increasing age, and the minority group showed an upward trend (*F* = 4.241, *P* = 0.002); At level 2 and the max, there were no significant ATR changes with age in the two groups (level 2: *F* = 0.740, *P* = 0.565; max: *F* = 2.155, *P* = 0.072). The ATR value of Han adolescents was greater at level 2 and max (level 2: *F* = 4.559, *P* = 0.033; max: *F* = 3.960, *P* = 0.047), especially at level 2 in those age 12 years (level 2, *Z* = −2.880, *P* = 0.004) and at the maximum level in those age 13 years (max, *Z* = −2.433, *P* = 0.015). At all measurement levels, ATR changed with age among Han participants, except for level 1; in minority participants, the interaction effects were statistically significant (age × ethnicity – level 1: *F* = 0.742, *P* = 0.563; level 2: *F* = 2.618, *P* = 0.033; level 3: *F* = 4.712, *P* = 0.001; max: *F* = 4.213, *P* = 0.002).

**Figure 2:**
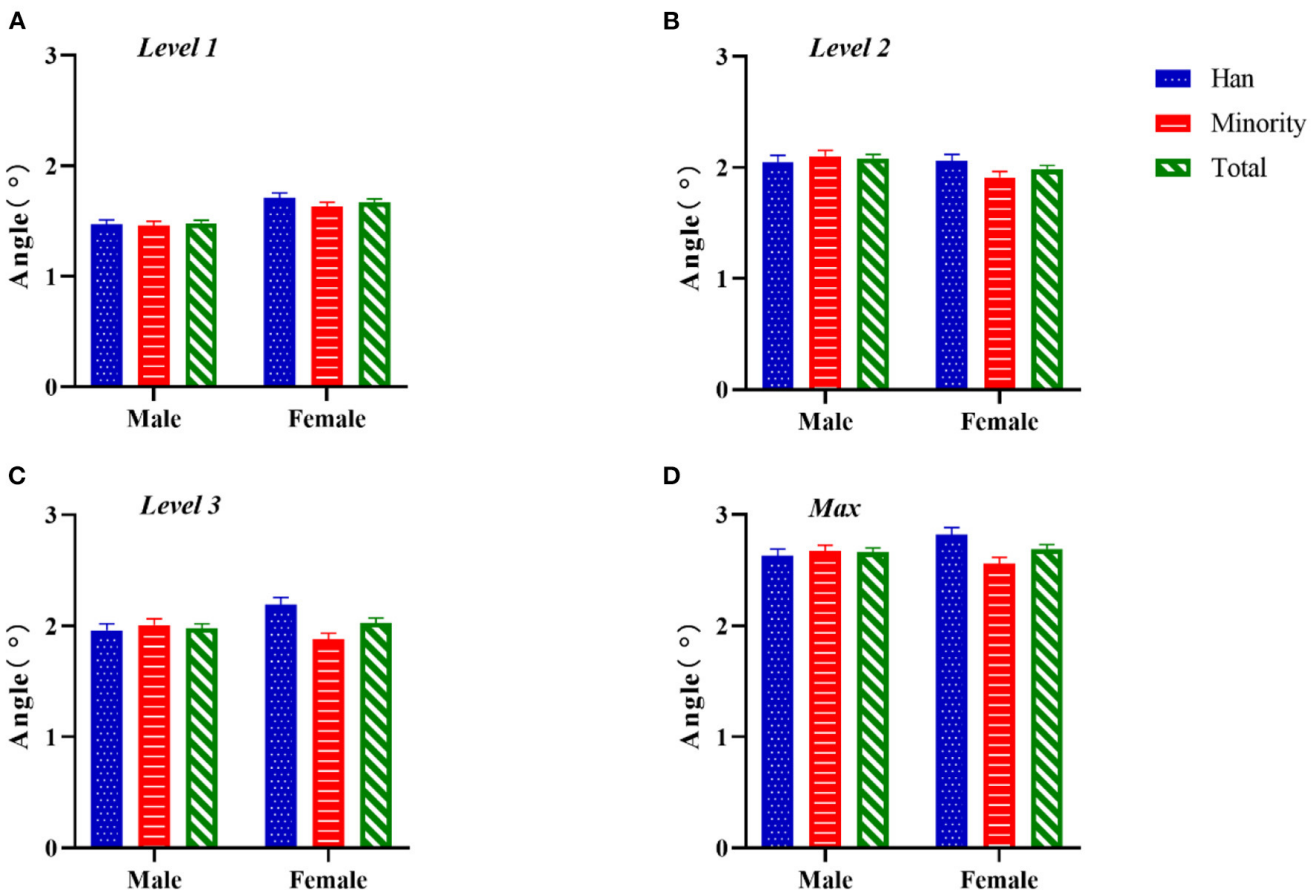
Two-factor analysis of variance to assess differences in ATR values by ethnicity and sex. **(A)** Level 1, proximal thoracic (T1–T4); **(B)** level 2, main thoracic (T5–T12); **(C)** level 3, lumbar (T12–L4); **(D)** Max, maximum value among the three levels.

As shown in Figure 2, the ATR values among girls were larger than those among boys, especially in Han girls (level 1: *Z* = −4.113, *P* < 0.0001; level 2: *Z* = −0.442, *P* = 0.658; level 3: *Z* = −2.719, *P* = 0.007; max: *Z* = −2.812, *P* = 0.005). The difference between ethnic groups was not significant, only at level 3 (*F* = 5.027, *P* = 0.025). The ATR values among Han girls were significantly higher than those among ethnic minorities (*Z* = −3.352, *P* = 0.001). There was non-significant interaction for region × sex, except at level 3 and max (level 1: *F* = 0.676, *P* = 0.411; level 2: *F* = 3.292, *P* = 0.070; level 3: *F* = 6.257, *P* = 0.012; max: *F* = 6.257, *P* = 0.012).

**Figure 3:**
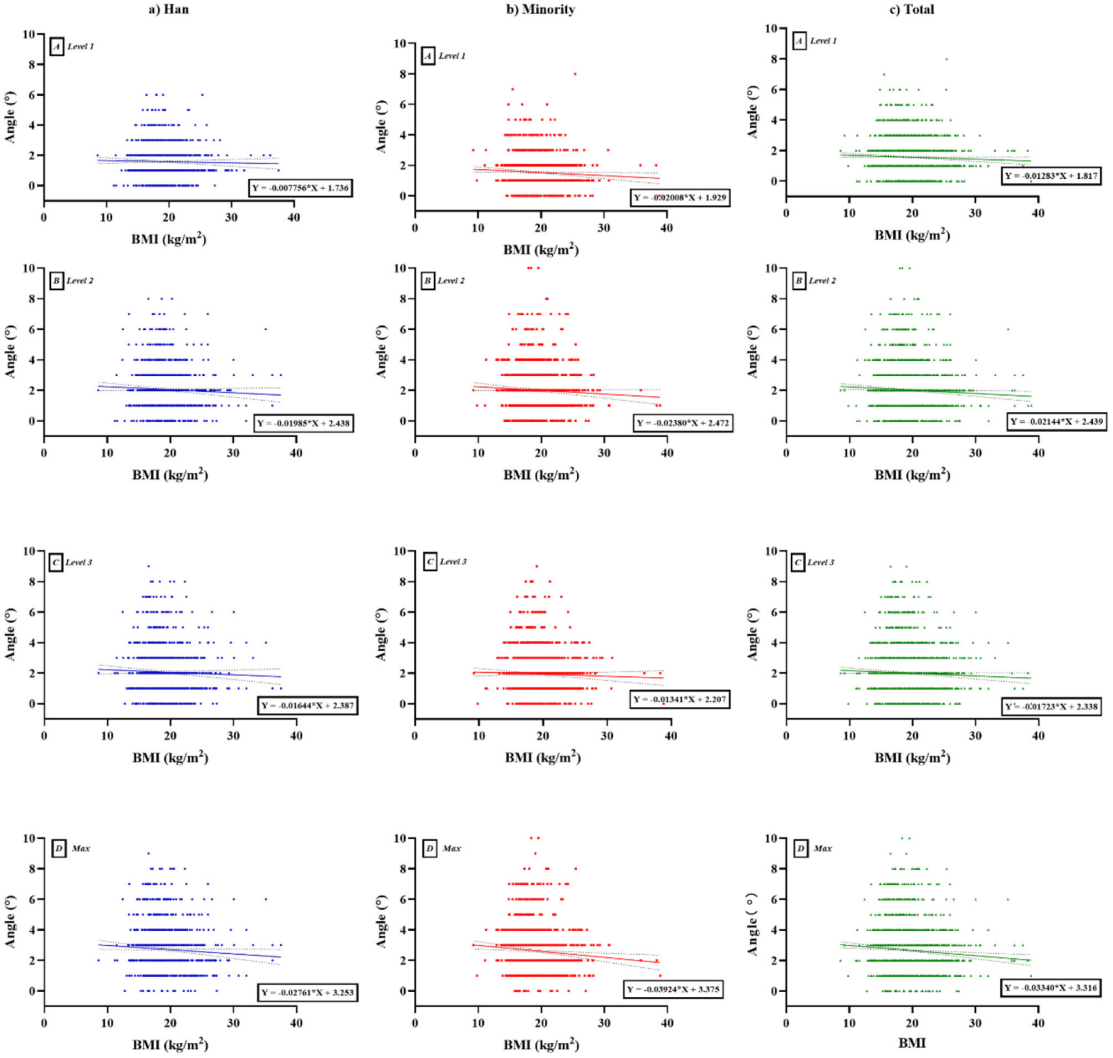
Scatter plot with trend line according to BMI and ATR between Han and ethnic minority groups. (A) Level 1, proximal thoracic (T1–T4); (B) level 2, main thoracic (T5–T12); (C) level 3, lumbar (T12–L4); (D) Max, maximum value among the three levels. **(a)** Han, **(b)** minority, **(c)** total. BMI, body mass index; ATR, angle of trunk rotation.

Figure 3 shows a weak negative correlation between ATR and BMI, which varied between the two groups (max: Han, *r* = −0.059, *P* = 0.044; ethnic minorities, *r* = −0.085, *P* = 0.002; Total, *r* = −0.074, *P* < 0.0001). At level 1, there was a significant negative correlation overall, but Han ethnicity alone was not statistically significant (level 1: Han, *r* = −0.023, *P* = 0.435; ethnic minorities, *r* = −0.061, *P* = 0.029; Total, *r* = −0.043, *P* = 0.034). At level 2, there was a significant negative correlation overall, but no statistical significance was observed for Han or ethnic minority groups alone (level 2: Han, *r* = −0.045, *P* = 0.124; ethnic minorities, *r* = −0.054, *P* = 0.054; Total, *r* = −0.050, *P* = 0.013). At level 3, there was no significant correlation between BMI and ATR for the two groups. Compared with the Han group, BMI had a greater impact on ATR among ethnic minorities.

**Figure 4:**
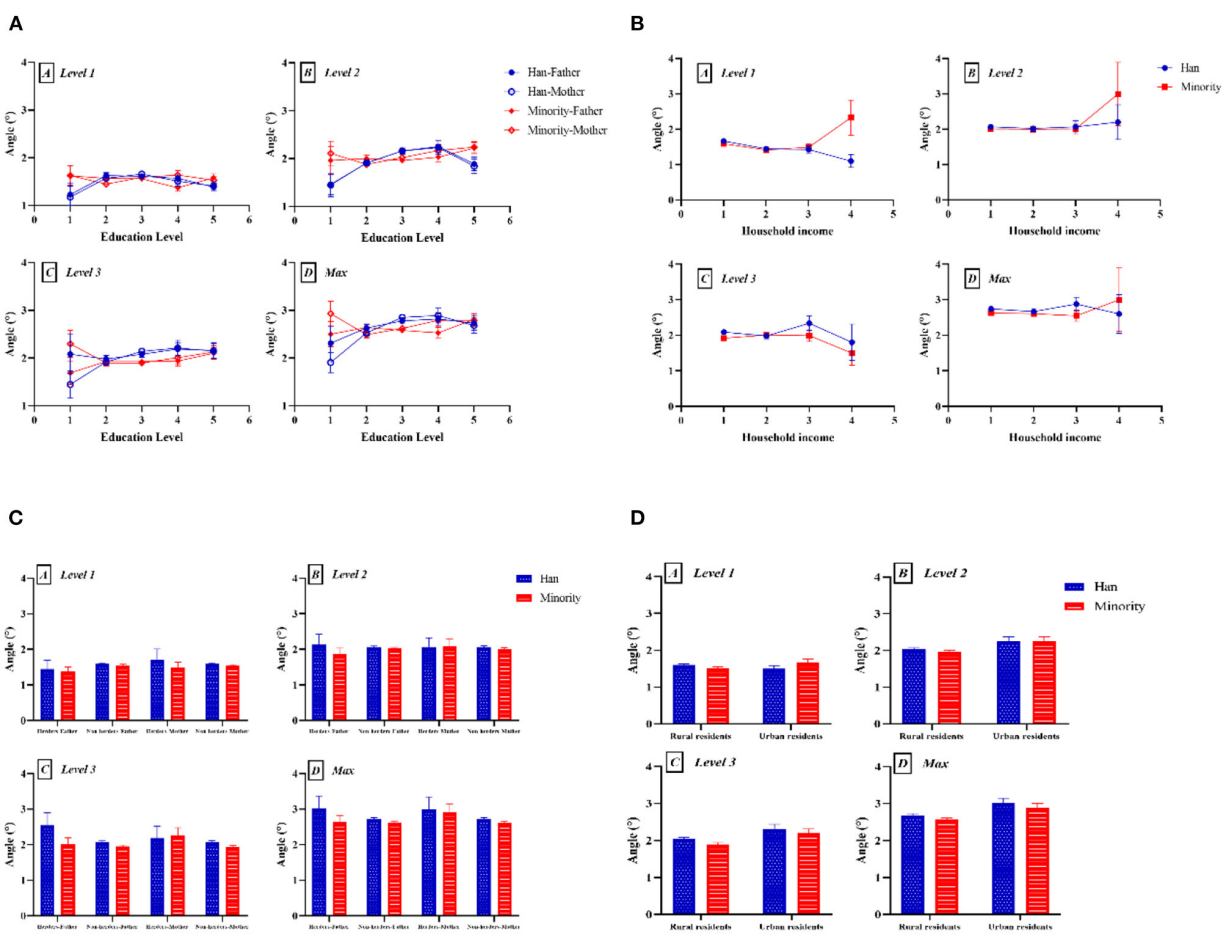
Two-factor analysis of variance to assess differences in ATR value by ethnicity and SES-related factors: **(A)** education level; **(B)** household income; **(C)** occupation; and **(D)** hukou. (A) Level 1, proximal thoracic (T1–T4); (B) level 2, main thoracic (T5–T12); (C) level 3, lumbar (T12–L4); (D) Max, maximum value among the three levels.

We analyzed SES-related variables and ethnicity, and the results are shown in Figure 4. The ATR values increased with an increase in the parents' educational level (Figure 4A), especially the mother's education level (level 2, *F* = 3.609, *P* = 0.006; max, *F* = 4.198, *P* = 0.002). As shown in Figure 4B, ATR values had no significant correlation with household income, except in level 1 (ethnicity: *F* = 4.265, *P* = 0.039; household income: *F* = 6.164, *P* < 0.0001; ethnicity × household income: *F* = 2.209, *P* = 0.085). Figure 4C shows that the ATR values for students whose parents are herders was relatively higher than those for students whose parents were non-herders, with no significant difference. As can be seen in Figure 4D, the ATR values among urban residents were significantly higher than those of rural residents (level 1: *F* = 0.291, *P* = 0.590; level 2: *F* = 10.974, *P* = 0.001; level 3: *F* = 11.990, *P* = 0.001; max: *F* = 15.375, *P* < 0.0001).

**Figure 5:**
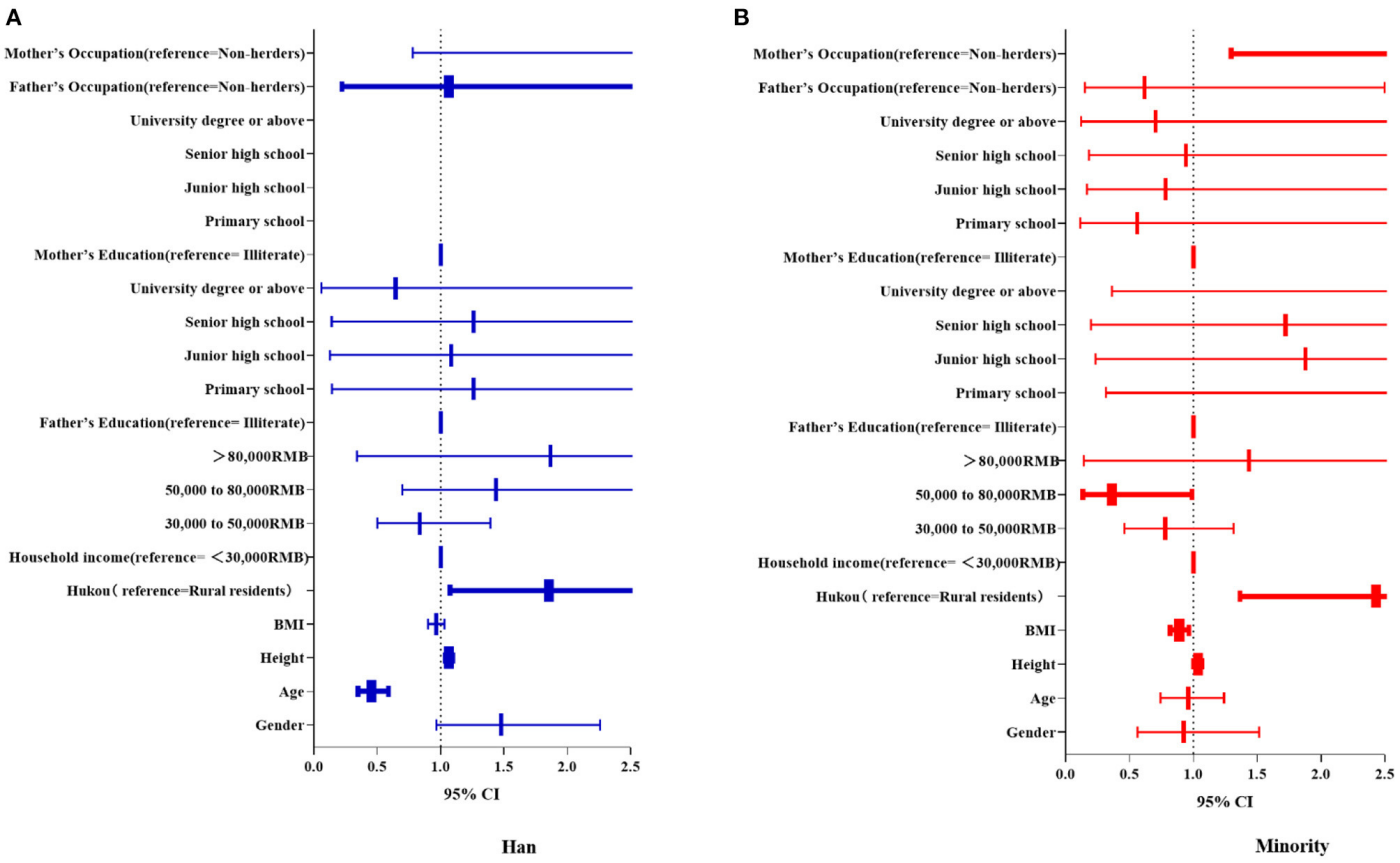
Forest map comparing differences in the influence of various factors on scoliosis between the two groups. Bold font indicates statistically significant variables. Sex, age, height, BMI, and SES-related factors were independent variables, and ATR value ≥ 5° was the dependent variable in the logistics regression model of **(A)** Han and **(B)** ethnic minority groups. BMI, body mass index; SES, socioeconomic status; ATR, angle of trunk rotation; CI, confidence interval.

The logistic regression model results are shown in Figure 5. From Figure 5A, we can see that height (OR 1.066, 95% CI, 1.037–1.096) and urban residence (OR 1.855, 95% CI, 1.074–3.206) were risk factors for the onset of scoliosis among Han students whereas age (OR 0.452, 95% CI, 0.347–0.589) was a protective factor. Figure 5B shows that height (OR 1.036, 95% CI, 1.004–1.068), urban residence (OR 2.432, 95% CI, 1.365–4.332), and mother's occupation as a herder (OR 5.046, 95% CI, 1.298–19.625) were risk factors for the incidence of scoliosis in ethnic minority students whereas BMI (OR 0.891, 95% CI, 0.822–0.966) and household income (50,000–80,000 RMB vs. <30,000 RMB: OR 0.363, 95% CI, 0.133–0.990) were protective factors. We clearly observed that participants' height and hukou were particularly important for the incidence of scoliosis.

## Discussion

In this study, for the first time, we showed that the positive rate for scoliosis is different between Han and ethnic minorities in western China, with Han adolescents having a higher positive rate of IS (10.8%) than ethnic minority adolescents (7.1%). One study pointed out that the Cobb angle corresponding to ATR = 5° was 10° (21). When ATR = 5° was used as the critical value, the sensitivity increased to 100%, but the specificity decreased to 47% (22). We suspect that the prevalence of scoliosis among Han students is 5.08% and that among ethnic minorities is 3.34% in the Tianzhu Tibetan Autonomous Region. Compared with previous research findings (0.11–2.64%), the prevalence rate of IS among both Han and ethnic minorities has increased slightly (15). Using the same screening tool, the results of SSS vary from country to country, with a positive IS rate of 4.57–15.9% and an IS prevalence of 0.76–2.55% (3, 23, 24). In terms of severity of scoliosis, the two groups were also different (Han, degree I = 8.1%, degree II = 2.7%, degree III = 0%; ethnic minorities, degree I = 4.5%, degree II = 2.3%, degree III = 0.3%). In degree III scoliosis, some abnormalities can be clearly seen from an observation of the body, such as uneven shoulders and scapular asymmetry. The proportion of ethnic minorities with degree III IS was higher than that of Han participants, which may be because parents do not pay much attention to their children's posture, which is contrary to existing research about ethnic disparities in self-rated health (14).

There was an interaction effect between ethnicity and age in this study. The ATR values among ethnic minority adolescents increased with age whereas this was the opposite among Han adolescents; this finding is very different from global reports regarding the change in ATR (18). Changes in education policies in relatively poor areas have caused academic pressure to increase each year, with younger children also experiencing heavier academic pressure (25). According to the policy in China, ethnic minority students receive bonus points when entering higher education, so the pressure on Han students is even greater (26). Han students may need to spend more time studying so as to stand out, leading to long hours seated at a desk, which can lead to abnormal spinal posture development (27).

The prevalence of IS is also linked to sex. IS is more prevalent in girls than in boys (28), and in this study, the ATR values among girls were greater than those among boys. For example, a Hong Kong cohort study found that during adolescence, for all disease severities, IS was more common in girls than in boys, with sex ratios of 2.7 for spinal curves of ≥10°, 4.5 for spinal curves of ≥20°, 8.1 for spinal curves of ≥40°, and 8.4 for spinal curves that needed treatment (29). We also found that the ATR values among Han girls were particularly high in comparison with those of ethnic minority girls at level 3, which may related to sedentary behavior, bone condition, and calcium intake owing to dietary pattern disparities (30, 31).

A meta-analysis by Tarrant et al. (32) showed that the BMI of patients with IS was significantly lower than that of healthy controls, which is consistent with the negative correlation between ATR and BMI obtained in our study. The relatively higher BMI of ethnic minority students may explain their lower incidence of scoliosis, which is related to dietary patterns (high energy and high protein) with a nomadic lifestyle (10). Dietary factors are also involved in the development of postural deformities like scoliosis (33). Lower BMI is a risk factor for both rehabilitation and surgical treatment in the later stages. Goodbody et al. (34) pointed out that compared with normal BMI, patients with excessively high (>85%) or excessively low (<20%) BMI showed less effective brace treatment, and low BMI was an independent risk factor for brace treatment failure. Similarly, the operation rate among patients with low BMI is higher than that of controls (35). Therefore, maintaining proper BMI is essential to reduce the risk of scoliosis. Additionally, some studies have pointed out that obese patients have a larger deformity curve than normal-weight patients with IS because the thicker cortex may mask the manifestations of spinal deformity in scoliometer screening (34, 36). Thus, it has been suggested that new referral criteria for screening obese children should be proposed (36).

SES factors are closely related to the incidence of diseases. The findings of this study were highly consistent with those of international research, especially with respect to the influence of education level, occupation, and hukou (37, 38). In this study, parents of children with relatively larger ATR values had higher education levels. Generally speaking, the higher the parent's educational level, the more attention will be paid to the child's studies, and the child will have more sedentary time spent studying (39). We found that the proximal thoracic ATR value of Han adolescents was negatively correlated with household income whereas it was positively correlated in ethnic minority students. One study pointed out two other dimensions, in which the main thoracic curve magnitude and the max magnitude were negatively correlated with family income (40), which is consistent with our findings. It may be because only wealthier families are able to visit a doctor to screen for scoliosis. Parents' occupation as a herder had a major impact on the onset of scoliosis in children of both groups. Children from these families are more likely to be exposed to activities such as horseback riding (41, 42). Studies have pointed out that equestrian sports can cause changes in the shape of the sagittal plane of the spine and can even lead to thoracolumbar injuries.

In this study, we also found that hukou was a significant factor in scoliosis development, and the ATR values of urban students were greater than those of rural students in both groups. The hukou system in China, which classifies each person as a rural or an urban resident, is a major means of controlling population mobility (43). The system gives preferential treatment to urban hukou in nearly every aspect, including education, job opportunities, housing, health insurance, and other social services and regulations (44). Generally speaking, children with urban residence have greater opportunities to receive health care services, which provide a greater likelihood of identifying IS earlier. However, for children with urban residence, the external environment may be unsuitable for engaging in outdoor sports, resulting in a decrease in their physical activity levels. At the same time, studies have shown that students with high SES have better academic performance (45), which implies more sitting time, which is detrimental to spine health.

### Strength and Limitation

This is the first study on the risk factors of scoliosis from the perspective of ethnicity in China. It is a pity that our research did not collect follow-up diagnosis and rehabilitation data, due to the inconvenience caused by the COVID-19 and the long distance.

## Conclusion

This study showed that Han students in Tianzhu Tibetan Autonomous County were more likely to have scoliosis than ethnic minority students. The differences may be owing to differences in growth and development, diet, SES, and other unknown factors. Further research is needed to clarify this difference, which will benefit the prevention of IS in vulnerable populations.

## Data Availability Statement

Owing to the private nature of spine health among adolescents in this study, the data are not publicly available but may be obtained from the corresponding author on reasonable academic request. Requests to access the datasets should be directed to: duqing@xinhuamed.com.cn.

## Ethics Statement

The studies involving human participants were reviewed and approved by the Ethics Committee of Xinhua Hospital Affiliated to Shanghai Jiao Tong University School of Medicine. Written informed consent to participate in this study was provided by the participants' legal guardian/next of kin.

## Author Contributions

HG and YY conducted the literature review, drafted the outline of the manuscript, contributed to the analysis, and interpretation. NC, XZ, XL, and YJ contributed to the overall design of the paper, led the analysis, interpretation of data, and contributed to the discussion and overall paper. JH and QD did critical revisions. All authors contributed to drafting, approved the final version for publication, and agreed to be accountable for all aspects of the work in ensuring that questions related to the accuracy or integrity of any part of the work are appropriately investigated and resolved.

## Funding

This work was funded by the General Program of National Natural Science Foundation of China (81972030), the Clinical Research Plan of SHDC (No. SHDC 2020CR3041B), Advanced and Appropriate Technology Promotion Projects of Shanghai Municipal Health Commission (2019SY021).

## Conflict of Interest

The authors declare that the research was conducted in the absence of any commercial or financial relationships that could be construed as a potential conflict of interest.

## Publisher's Note

All claims expressed in this article are solely those of the authors and do not necessarily represent those of their affiliated organizations, or those of the publisher, the editors and the reviewers. Any product that may be evaluated in this article, or claim that may be made by its manufacturer, is not guaranteed or endorsed by the publisher.

## References

[1] Zavatsky JM Peters AJ Nahvi FA Bharucha NJ Trobisch PD Kean KE. Disease severity and treatment in adolescent idiopathic scoliosis: the impact of race and economic status. Spine J. (2015) 15:939–43. 10.1016/j.spinee.2013.06.043 24099683

[2] Hresko MT Talwalkar V Schwend R. Early detection of idiopathic scoliosis in adolescents. JBJS. (2016) 98:e67. 10.2106/JBJS.16.00224 27535448

[3] Yilmaz H Zateri C Kusvuran Ozkan A Kayalar G Berk H. Prevalence of adolescent idiopathic scoliosis in Turkey: an epidemiological study. Spine J. (2020) 20:947–55. 10.1016/j.spinee.2020.01.008 31972303

[4] Weinstein SL Dolan LA Spratt KF Peterson KK Spoonamore MJ Ponseti IV. Health and function of patients with untreated idiopathic scoliosis: a 50-year natural history study. Jama. (2003) 289:559–67. 10.1001/jama.289.5.559 12578488

[5] Alamrani S Rushton A Gardner A Falla D Heneghan NR. Outcome measures evaluating physical functioning and their measurement properties in adolescent idiopathic scoliosis: a protocol for a systematic review. BMJ Open. (2020) 10:e034286. 10.1136/bmjopen-2019-034286 32241788 PMC7170637

[6] Grivas TB Wade MH Negrini S O'Brien JP Maruyama T Hawes MC. SOSORT consensus paper: school screening for scoliosis. Where are we today? Scoliosis. (2007) 2:17. 10.1186/1748-7161-2-17 18039374 PMC2228277

[7] Altaf F Drinkwater J Phan K Cree AK. Systematic review of school scoliosis screening. Spine Deform. (2017) 5:303–9. 10.1016/j.jspd.2017.03.009 28882347

[8] Lei YT Ma J Hu PJ Dong B Zhang B Song Y. The status of spermarche, menarche and corresponding relationships with nutritional status among students of 13 ethnic minorities in Southwest China in 2014. Zhonghua Yu Fang Yi Xue Za Zhi. (2019) 53:492–6. 10.3760/cma.j.issn.0253-9624.2019.05.011 31091607

[9] Ruan Y Huang Y Zhang Q Qin S Du X Sun Y. Association between dietary patterns and hypertension among Han and multi-ethnic population in southwest China. BMC Public Health. (2018) 18:1106. 10.1186/s12889-018-6003-7 30200909 PMC6131804

[10] Lek N Yan W Zhang Y Wang Q Cheung YB. Indices of central and general obesity and cardiometabolic risk among adolescents in three ethnic groups in north-west China. Ann Hum Biol. (2016) 43:18–24. 10.3109/03014460.2015.1014418 26431471

[11] Liu M Zhang Q Lu M Kwon CS Quan H. Rural and urban disparity in health services utilization in China. Med Care. (2007) 45:767–74. 10.1097/MLR.0b013e3180618b9a 17667311

[12] He J Assanangkornchai S Cai L McNeil E. Disparities in drinking patterns and risks among ethnic majority and minority groups in China: the roles of acculturation, religion, family and friends. Drug Alcohol Depend. (2016) 159:198–206. 10.1016/j.drugalcdep.2015.12.028 26790824

[13] Chen P He G Zou X Zhang X Li J Wang Z. Genetic diversities and phylogenetic analyses of three Chinese main ethnic groups in southwest China: a Y-Chromosomal STR study. Sci Rep. (2018) 8:15339. 10.1038/s41598-018-33751-x 30337624 PMC6193932

[14] Wang YJ Chen XP Chen WJ Zhang ZL Zhou YP Jia Z. Ethnicity and health inequalities: an empirical study based on the 2010 China survey of social change (CSSC) in Western China. BMC Public Health. (2020) 20:637. 10.1186/s12889-020-08579-8 32380963 PMC7204236

[15] Zhang H Guo C Tang M Liu S Li J Guo Q. Prevalence of scoliosis among primary and middle school students in Mainland China: a systematic review and meta-analysis. Spine (Phila Pa 1976). (2015) 40:41–9. 10.1097/BRS.0000000000000664 25341979

[16] Yang Q Feng GY Xiao XH. The epidemiologic investigation and research of pupils' spine lateral curvature on Baiyin city. Chin J Pract Med. (2005) 5:7.

[17] Di JY Xing B Lei GF Fang XQ Zhang CG. Surveillance and analysis of common diseases among primary and middle school students in Gaotai County in 2019. Dis Prev Control Bull. (2020) 35:70–2+5. 10.13215/j.cnki.jbyfkztb.2005017

[18] Adamczewska K Wiernicka M Malchrowicz-Mośko E Małecka J Lewandowski J. The angle of trunk rotation in school children: a study from an idiopathic scoliosis screening. Prevalence and optimal age screening value. Int J Environ Res Public Health. (2019) 16:3426. 10.3390/ijerph16183426 31527403 PMC6765789

[19] Negrini S Donzelli S Aulisa AG Czaprowski D Schreiber S de Mauroy JC. 2016 SOSORT guidelines: orthopaedic and rehabilitation treatment of idiopathic scoliosis during growth. Scoliosis Spinal Disord. (2018) 13:3. 10.1186/s13013-017-0145-8 29435499 PMC5795289

[20] GB/T16133-−2014. Screening of Spinal Curvature Abnormality of Children and Adolescents[S]. Beijing: National Health and Family Planning Commission of the People's Republic of China, China National Standardization Management Committee (2015).

[21] Coelho DM Bonagamba GH Oliveira AS. Scoliometer measurements of patients with idiopathic scoliosis. Braz J Phys Ther. (2013) 17:179–84. 10.1590/S1413-35552012005000081 23778766

[22] Ashworth MA Hancock JA Ashworth L Tessier KA. Scoliosis screening. An approach to cost/benefit analysis. Spine (Phila Pa 1976). (1988) 13:1187–8. 10.1097/00007632-198810000-00024 3144755

[23] Deepak AS Ong JY Choon D Lee CK Chiu CK Chan C. The clinical effectiveness of school screening programme for idiopathic scoliosis in Malaysia. Malays Orthop J. (2017) 11:41–6. 10.5704/MOJ.1703.018 28435573 PMC5393113

[24] Aulisa AG Giordano M Guzzanti V Falciglia F Pizzetti P Toniolo RM. Effectiveness of school scoliosis screening and the importance of this method in measures to reduce morbidity in an Italian territory. J Pediatr Orthop B. (2019) 28:271–7. 10.1097/BPB.0000000000000611 30807511

[25] The State Council. Guiding Opinions of the General Office of the State Council on Accelerating the Development of Education in the Midwest. (2016). Available online at: http://www.gov.cn/zhengce/content/2016-06/15/content_5082382.htm (accessed April 12, 2022).

[26] Ministry of Education of the People's Republic of China. Regulations on Admissions for Regular Colleges and Universities in 2019. (2019). Available online at: http://www.moe.gov.cn/srcsite/A15/moe_776/s3258/201904/t20190415_378127.html (accessed April 12, 2022).

[27] Tobias JH Fairbank J Harding I Taylor HJ Clark EM. Association between physical activity and scoliosis: a prospective cohort study. Int J Epidemiol. (2019) 48:1152–60. 10.1093/ije/dyy268 30535285 PMC6896242

[28] Cheng JC Castelein RM Chu WC Danielsson AJ Dobbs MB Grivas TB. Adolescent idiopathic scoliosis. Nat Rev Dis Primers. (2015) 1:15030. 10.1038/nrdp.2015.30 27188385

[29] Luk KD Lee CF Cheung KM Cheng JC Ng BK Lam TP. Clinical effectiveness of school screening for adolescent idiopathic scoliosis: a large population-based retrospective cohort study. Spine (Phila Pa 1976). (2010) 35:1607–14. 10.1097/BRS.0b013e3181c7cb8c 20453727

[30] Chopra S Larson AN Kaufman KR Milbrandt TA. Accelerometer based assessment of daily physical activity and sedentary time in adolescents with idiopathic scoliosis. PLoS ONE. (2020) 15:e0238181. 10.1371/journal.pone.0238181 32877408 PMC7467220

[31] Lee WT Cheung CS Tse YK Guo X Qin L Ho SC. Generalized low bone mass of girls with adolescent idiopathic scoliosis is related to inadequate calcium intake and weight bearing physical activity in peripubertal period. Osteoporos Int. (2005) 16:1024–35. 10.1007/s00198-004-1792-1 15726296

[32] Tarrant RC Queally JM Moore DP Kiely PJ. Prevalence and impact of low body mass index on outcomes in patients with adolescent idiopathic scoliosis: a systematic review. Eur J Clin Nutr. (2018) 72:1463–84. 10.1038/s41430-018-0095-0 29434317

[33] Bammann K Peplies J Pigeot I Ahrens W. IDEFICS: a multicenter European project on diet- and lifestyle-related disorders in children. Med Klin (Munich). (2007) 102:230–5. 10.1007/s00063-007-1027-2 17345019

[34] Goodbody CM Asztalos IB Sankar WN Flynn JM. It's not just the big kids: both high and low BMI impact bracing success for adolescent idiopathic scoliosis. J Child Orthop. (2016) 10:395–404. 10.1007/s11832-016-0763-3 27501808 PMC5033782

[35] Karol LA Wingfield JJ Virostek D Felton K Jo C. The influence of body habitus on documented brace wear and progression in adolescents with idiopathic scoliosis. J Pediatr Orthop. (2020) 40:e171–e5. 10.1097/BPO.0000000000001420 31259783

[36] Margalit A McKean G Constantine A Thompson CB Lee RJ Sponseller PD. Body mass hides the curve: thoracic scoliometer readings vary by body mass index value. J Pediatr Orthop. (2017) 37:e255–e60. 10.1097/BPO.0000000000000899 27861214 PMC5422115

[37] Link BG Phelan J. Social conditions as fundamental causes of disease. J Health Soc Behav. (1995) 36:80–94. 10.2307/2626958 7560851

[38] White K. The sustaining relevance of W.E.B.DU BOIS to health disparities research. Du Bois Rev Soc Sci Res Race. (2011) 8:285–93. 10.1017/S1742058X11000233

[39] Lu H Nie P Sousa-Poza A. The effect of parental educational expectations on adolescent subjective well-being and the moderating role of perceived academic pressure: longitudinal evidence for China. Child Ind Res. (2021) 14:117–37. 10.1007/s12187-020-09750-8

[40] Gilbert SR Savage AJ Whitesell R Conklin MJ Fineberg NS. BMI and magnitude of scoliosis at presentation to a specialty clinic. Pediatrics. (2015) 135:e1417–24. 10.1542/peds.2014-2000 25963009

[41] Ginés-Díaz A Martínez-Romero MT Cejudo A Aparicio-Sarmiento A Sainz de Baranda P. Sagittal spinal morphotype assessment in dressage and show jumping riders. J Sport Rehabil. (2020) 29:533–40. 10.1123/jsr.2018-0247 31034307

[42] Schröter C Schulte-Sutum A Zeckey C Winkelmann M Krettek C Mommsen P. Accidents in equestrian sports: analysis of injury mechanisms and patterns. Unfallchirurg. (2017) 120:129–38. 10.1007/s00113-015-0074-z 26449915

[43] Chan KW. The household registration system and migrant labor in China: notes on a debate. Popul Dev Rev. (2010) 36:357–64. 10.1111/j.1728-4457.2010.00333.x 20734556

[44] Song Q Smith JP. Hukou system, mechanisms, and health stratification across the life course in rural and urban China. Health Place. (2019) 58:102150. 10.1016/j.healthplace.2019.102150 31212169 PMC6708454

[45] Shore E Cheung PC Hyde E Gazmararian JA. Physical activity opportunities and academic outcomes of fourth grade elementary school students in Georgia. J Sch Health. (2020) 90:25–31. 10.1111/josh.12846 31770813

